# SARS-CoV-2 Dynamics in the Premier League Testing Program, United Kingdom

**DOI:** 10.3201/eid3009.240853

**Published:** 2024-09

**Authors:** Adam J. Kucharski, Timothy W. Russell, Joel Hellewell, Sebastian Funk, Andrew Steele, W. John Edmunds, Mark Gillett

**Affiliations:** London School of Hygiene & Tropical Medicine, London, UK (A.J. Kucharski, T.W. Russell, S. Funk, W.J. Edmunds);; European Bioinformatics Institute at Wellcome Genome Campus, Hinxton, UK (J. Hellewell);; Stride Health Group, London (A. Steele);; The Football Association Premier League, London (M. Gillett)

**Keywords:** COVID-19, SARS-CoV-2, severe acute respiratory syndrome coronavirus 2, viruses, respiratory infections, zoonoses, English Premier League, PCR testing, infection prevalence, United Kingdom

## Abstract

During 2020–2022, players and staff in the English Premier League in the United Kingdom were tested regularly for SARS-CoV-2 with the aim of creating a biosecure bubble for each team. We found that prevalence and reinfection estimates were consistent with those from other studies and with community infection trends.

During the COVID-19 pandemic, frequent SARS-CoV-2 testing regardless of symptoms was used to reduce transmission risk in several workplace populations (*1*,*2*). In the process, data from those cohorts generated a range of scientific insights, including estimates of viral shedding dynamics and patterns of immune responses against novel variants of concern. Such testing programs in different settings are valuable for informing planning for future pandemics, especially given likely variation in behavior and therefore risk across subpopulations. Sports testing programs pose a particular challenge for infection control given the potential number of contacts made within and between teams and their support staff.

English Premier League (EPL) fixtures were suspended on March 19, 2020; such events included not only the games but also related social and behavioral aspects, such as fan mingling outside the venues and at concession stands. In May, a strategy was developed to allow games to resume while minimizing transmission risk, including not allowing fan attendance. A key principle of this strategy was a biosecure bubble for each team, which involved SARS-CoV-2 testing of core players and staff twice a week. Initially, PCR testing was used; during June‒November 2021 and from January 2022 on, rapid antigen testing was used for screening (regular PCR was used during the Omicron wave in December 2021), and PCR testing was performed only after a positive rapid test result (Appendix). For this study, we examined PCR test results recorded during May 11, 2020–March 2, 2022.

During the study period, 178,588 ​PCR tests were obtained from 7,552 unique persons. Throughout late 2020 and early 2021, and during the early stages of the Omicron wave in late 2021, SARS-CoV-2 PCR positivity in the EPL broadly mirrored positivity in the UK Office for National Statistics Community Infection Survey (S. Abbott et al., unpub. data, https://doi.org/10.1101/2022.03.29.22273101) among 16–24- and 25–29-year age groups (Figure, panel A). One exception was early outbreaks in August 2020, as well as a period in early 2021 where prevalence in the EPL program was lower than community prevalence in the Community Infection Survey.

**Figure:**
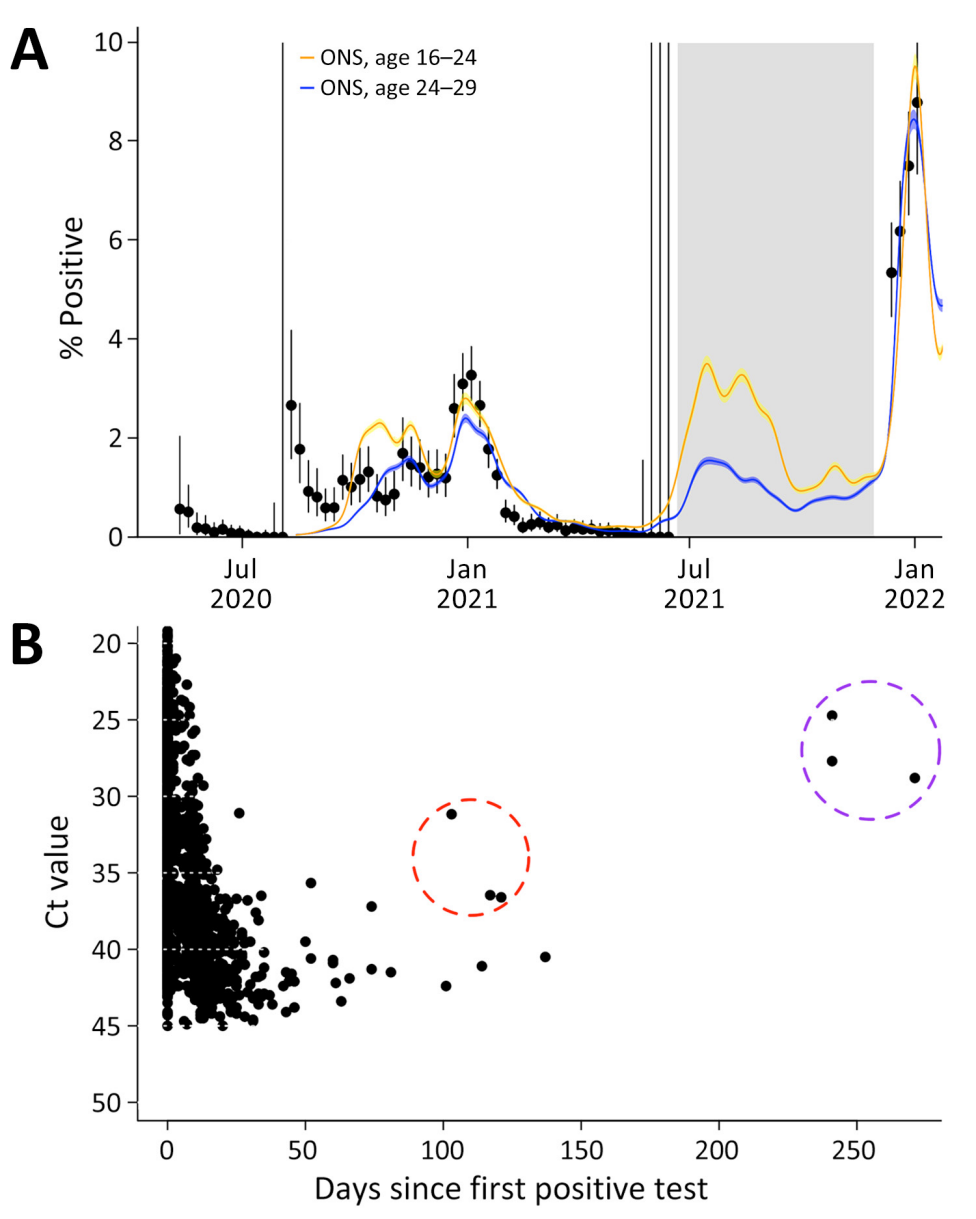
Infection dynamics in the English Premier League testing program for SARS-CoV-2, 2020–2022. A) Weekly PCR test positivity. Dots indicate infections; error bars indicate 95% CIs. Colored lines show inferred mean prevalence in the UK ONS Community Infection Survey. Gray shading shows period where the testing protocol was based on a rapid antigen test, with subsequent confirmatory PCR; hence, PCR positivity is not comparable because sampling was nonrandom. B) Distribution of individual Ct values over time since first positive test. Dashed circles indicate estimated reinfections: orange represents Alpha wave, in which 1 person was reinfected, as determined from 3 samples; purple represents Delta wave, in which samples indicate that 3 persons were reinfected. Ct, cycle threshold; ONS, Office for National Statistics.

Regular testing can also provide insights into epidemiologic characteristics such as reinfection risk and viral shedding. Several persons recorded positive PCR tests for several weeks after their initial positive test; we identified 1 person with evidence of a reinfection during the Alpha wave (indicated by cycle threshold values >40 after 90 days postinfection) and another 3 persons with evidence of reinfection during the Delta wave (Figure, panel B). Those findings reinforce the importance of effective communication in ensuring appropriate interpretation of results, particularly if persons are being tested regardless of symptoms; a single positive PCR test does not necessarily indicate a recent infection, but a test conducted after symptom onset does. The estimated odds ratio for subsequent infection during the Alpha wave among those infected before December 1, 2020, was therefore 0.1 (95% CI 0.004–0.4), which is broadly consistent with other cohort studies that have estimated ≈85% risk reduction following an initial infection (*3*). We also found that cycle threshold values at time of first positive test were higher on average during the pre-Alpha period (mean value 33.7, SD 6; n = 106) than during the Alpha wave (mean 29.2, SD = 5.8; n = 223), reflecting the increased shedding for Alpha that has been observed in other studies (S. Funk et al., unpub. data). 

In conclusion, we found that SARS-CoV-2 infection prevalence and reinfection estimates among EPL players and staff were consistent with those from other studies and with community infection trends. Such real-time insights could be further enhanced for future outbreaks or pandemics by adding sequencing or antibody testing.

Appendix Additional information about the English Premier League Testing Program for SARS-CoV-2.
