# Supplementary material for: SARS-CoV-2 Dynamics in the Premier League Testing Program, United Kingdom
Source: Emerg Infect Dis. 2024 Sep;30(9):1975–7. doi: 10.3201/eid3009.240853 (PMC11346986; doi:10.3201/eid3009.240853)
Supplement: Appendix — Additional information about the English Premier League Testing Program for SARS-CoV-2. [file 24-0853-Techapp-s1.pdf]

Article DOI: https://doi.org/10.3201/eid3009.240853

EID cannot ensure accessibility for supplementary materials supplied by authors. Readers who have difficulty accessing supplementary content should contact the authors for assistance.

SARS-CoV-2 Dynamics in the English Premier League Testing Program

Appendix

Description of Testing Program

During the testing period over 250,000 tests were carried out across both PCR and lateral flow tests (LFTs). Clubs were required to designate a set group of individuals who would form part of their core first team or match-day operations, who underwent repeated longitudinal testing during the program. Any individuals that had to join the training ground bubble would enter the testing program before they could be admitted – for example, if a player from an academy were temporarily brought in to the first team to cover for an injured player. Routine PCR screening continued until July 22nd 2021, at which point the testing program moved to regular LF testing (save for a temporary move back to regular PCR during the Omicron wave in December 2021). In response to the changing stages of the pandemic, the program moved to symptomatic testing only on April 4th 2022. During the symptomatic only testing phase, Clubs held stock of approved program LFT on site and upon presentation of symptoms the Club administered the tests directly. Any positive LFT cases were then externally confirmed with PCR via Prenetics.

The program continued until the end of Season 21/22. Because the program was designed for infection control rather than research, additional metadata beyond date and type of test were not available for analysis. PCR tests were performed with the Hologic Panther platform using the SARS-CoV-2TMA assay; this did not include an S-gene target, so it was not possible to distinguish between variants by S-gene dropout.

All samples were professionally administered by trained sample collectors or nurses, using anterior nasal swabbing for both PCR and LFT. PCR samples were collected and stored

until the end of a given testing session. Samples were then couriered to a central laboratory at the close of each testing session.

Transport times from testing site to central laboratory varied due to location, with the shortest transport at ~30 minutes and the longest at ~240 minutes. Across all testing locations, PCR samples were analyzed and resulted in an average time of 19 hours and 6 minutes from sample collection to result delivery, and with an average time of 16 hours and 16 minutes from receipt at the central laboratory to result delivery. The average sample storage, collection, transport and analysis protocol was consistent across the period.

LFT testing was performed using the SureScreen Diagnostics SARS-CoV-2 Antigen Rapid Test Cassette. Samples were professional collected and matured in a designated environment within each testing site, and results were interpreted by trained sample collectors or nurses with specific training on the LFT device being used.

Ethics Statement

Secondary analysis of the routinely collected testing data was approved by the LSHTM Observational Research Ethics Committee (ref 25204).

Odds of Reinfection

Prior to 1st December 2020, 165 individuals had at least one positive PCR test recorded and 3957 had no positive tests recorded. One individual was identified as a reinfection during the Alpha wave (i.e., defined as a Ct value above 40 in a sample more than 90 days after the original infection, with this reinfection detected during the period from 1st December 2021 to 1st April 2021), with 263 infections among the previously negative group. The resulting odds ratio, calculated using median-unbiased estimation with *oddsratio()* in the {epitools} R package (*1*), was therefore 0.1 (95% CI: 0.004–0.4).

Note that some infections may have been missed before the testing program began, meaning they would incorrectly have been categorised as ‘never infected’ in the above analysis. If these individuals were generally not among those infected during the Alpha wave, then it would reduce the odds ratio; if, however, several of them were among the subsequent infections,

it would increase the estimate of the odds ratio. In future, such uncertainty could be reduced with additional antibody testing at the start of a program and/or routine testing from the very early stages of the domestic epidemic.

Ct Values

Comparing the wildtype period of sampling (defined as before 1st Nov 2020, to avoid the period of co-circulation of both Alpha and wildtype) compared to the Alpha wave (defined as 1st Jan 2021 to 1st April 2021), we observed a lower distribution of Ct values, representing less viral shedding during the wildtype wave (Appendix Figure 1). Although such distributions can be influenced by the epidemic phase in cross-sectional sampling (*2*), the longitudinal study design, combined with periods of growth during both waves, reduces the possibility of such results being a statistical artifact only of the epidemic dynamics.

Comparison of Individual-Level PCR and LFT Positivity during Omicron Wave

During the early Omicron wave in December 2021, individuals were routinely tested by both LFT and PCR. This enabled direct comparison of the proportion who tested positive at different points in their infection (Appendix Figure 2). We estimated that the area under the curve for PCR positivity, denoted P, was around p = 6.9, compared to around p = 2.5 for the LFT curve, similar to the difference observed in an earlier study of test positivity among healthcare workers (*3*). Under the assumption that incidence is relatively consistent over the average duration of positivity, we can approximate incidence, I, from prevalence, P, using the formula $I = P / D$. Previous work has shown that this is a reasonable approximation with relatively small error for periods of fluctuating COVID dynamics over several weeks (*4*), although in situations where epidemic dynamics change rapidly within a week, it may be more appropriate to use inference methods that reconstruct incidence using an underlying generative model of the epidemic process (S. Abbott et al., unpub. data, https://doi.org/10.1101/2022.03.29.22273101).

The above approximation suggests that for a prevalence of 1% in a cross-sectional sample of LFT tests and an equivalent prevalence in PCR tests, the true incidence of daily infections would be around 0.01/2.5 = 0.4% in the LFT study and 0.01/6.9 = 0.15% in the PCR study.

Data and Code Availability

Code and aggregated data to reproduce the figures in this paper are available from: https://github.com/adamkucharski/pl-testing

References

1. Aragon T, et al. epitools: Epidemiology Tools 10.1, 2020 [cited 2024 Jul 25]. https://CRAN.R-project.org/package=epitools

2. Hay JA, Kennedy-Shaffer L, Kanjilal S, Lennon NJ, Gabriel SB, Lipsitch M, et al. Estimating epidemiologic dynamics from cross-sectional viral load distributions. Science. 2021;373:eabh0635. PubMed https://doi.org/10.1126/science.abh0635

3. Hellewell J, Russell TW, Beale R, Kelly G, Houlihan C, Nastouli E, et al.; SAFER Investigators and Field Study Team; Crick COVID-19 Consortium; CMMID COVID-19 working group. Estimating the effectiveness of routine asymptomatic PCR testing at different frequencies for the detection of SARS-CoV-2 infections. BMC Med. 2021;19:106. PubMed https://doi.org/10.1186/s12916-021-01982-x

4. Kucharski AJ, Chung K, Aubry M, Teiti I, Teissier A, Richard V, et al. Real-time surveillance of international SARS-CoV-2 prevalence using systematic traveller arrival screening: An observational study. PLoS Med. 2023;20:e1004283. PubMed https://doi.org/10.1371/journal.pmed.1004283

Appendix Table. SARS-CoV-2 reinfection in the English Premier League, 2020–2022

Category	Reinfection between 1st Dec 2020 and 1st April 2021	No reinfection between 1st Dec 2020 and 1st April 2021
At least one PCR positive before 1st Dec 2020	1	164
No PCR positive before 1st Dec 2020	263	3694

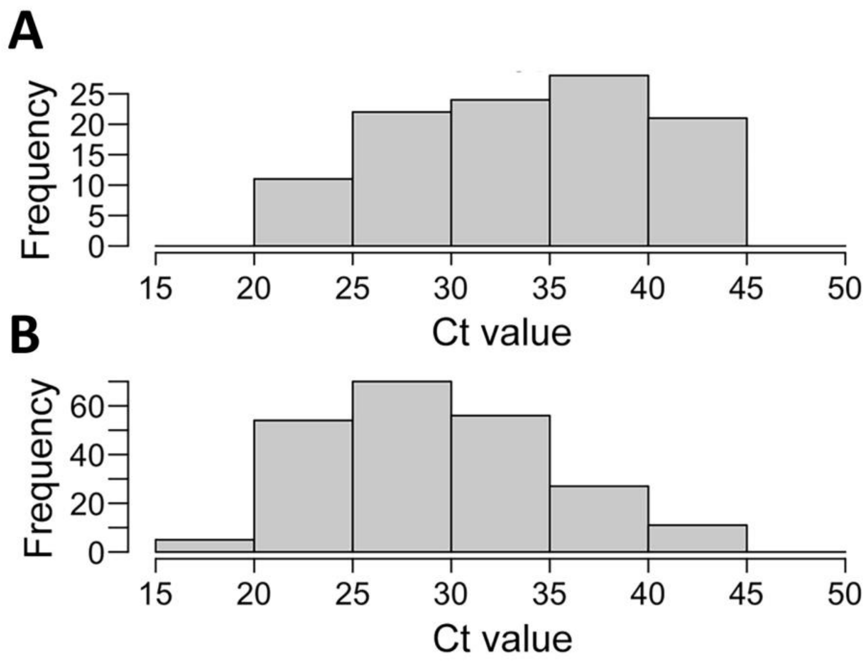

Appendix Figure 1. Distribution of Ct values among positive tests during the A) wildtype and B) Alpha waves.

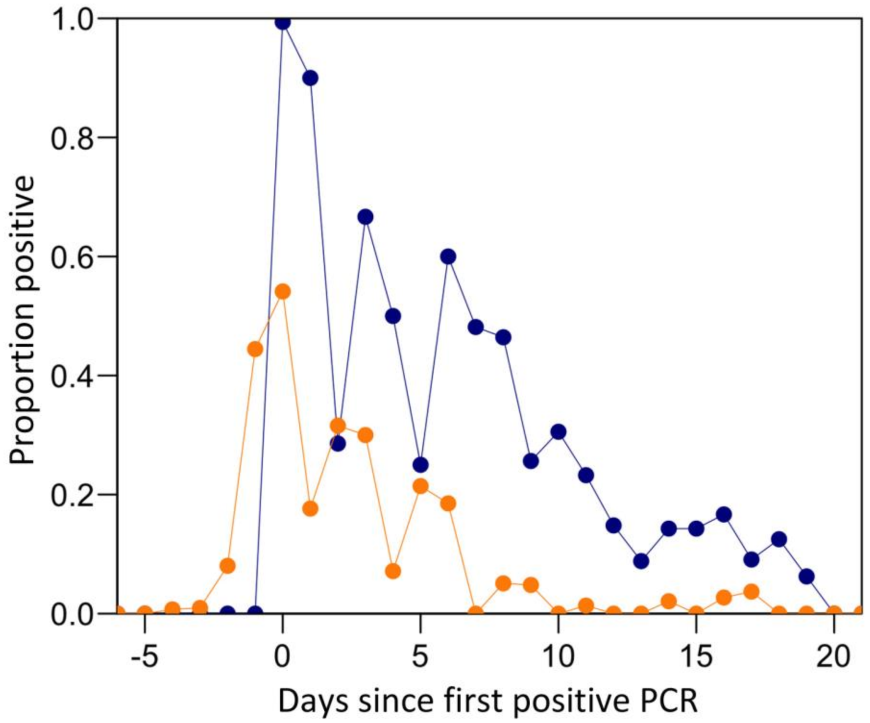

Appendix Figure 2. Proportion of individuals testing positive by PCR (blue dots) and LFT (orange dots), by time since their first positive PCR test. Note that there are no values below zero for the blue curve because by definition it is not possible to test positive by PCR before the first positive PCR test.
